# Mixed Lineage Kinase Domain‐Like Protein (MLKL): From Mechanisms to Therapeutic Opportunities

**DOI:** 10.1002/advs.202509277

**Published:** 2025-08-11

**Authors:** Lijuan Xu, Chunlin Zhuang

**Affiliations:** ^1^ The Center for Basic Research and Innovation of Medicine and Pharmacy (MOE) School of Pharmacy Naval Medical University/Second Military Medical University 325 Guohe Road Shanghai 200433 China; ^2^ School of Pharmacy Ningxia Medical University 1160 Shengli Street Yinchuan Ningxia 750004 China

**Keywords:** activation mechanisms, pseudokinase domain, MLKL, MLKL inhibitors, 4HB

## Abstract

Lytic forms of regulated cell death (RCD) rely on the activation and recruitment of executioner proteins. The mixed lineage kinase domain‐like protein (MLKL) acts as the executioner in the necroptosis pathway, transitioning from an inactive to active state through phosphorylation, oligomerization, membrane recruitment, and membrane insertion, ultimately forming membrane hotpots. These mechanisms involve protein–protein interactions between receptor‐interacting protein kinase 3 (RIPK3) and MLKL, MLKL phosphorylation, protein–protein interactions between MLKL and MLKL, and MLKL‐lipid interactions. In this review, the specificity of MLKL activation mechanisms is discussed across different species and describe the processes by which MLKL transitions from an auto‐inhibited to a membrane‐embedded state. The opportunities are further explored for targeting MLKL, including small molecule inhibitors and functionally interacting proteins.

## Graphical abstract

The mixed lineage kinase domain‐like protein (MLKL) acts as the executioner in the necroptosis pathway, transitioning from an inactive to active state through phosphorylation, oligomerization, membrane recruitment, and membrane insertion, ultimately forming membrane hotpots. In this review, the specificity of MLKL activation mechanisms is discussed across different species and describe the processes by which MLKL transitions from an auto‐inhibited to a membrane‐embedded state.

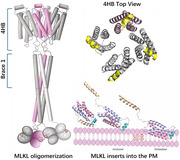

## 1 Introduction

The mixed lineage kinase domain‐like protein (MLKL) was first reported as a downstream target of receptor‐interacting protein kinase 1 (RIPK1) and RIPK3 in the necroptosis pathway.^[^ ^1^ ^]^ This was achieved through the use of MLKL inhibitor necrosulfonamide (NSA)‐derived affinity probes and anti‐RIPK3 antibody involved co‐immunoprecipitation. MLKL is reported not only to act as an executioner of the necroptosis pathway but also to participate in different forms of RCD pathways. For example, Pyroptosis, a type of RCD, is triggered by nucleotide‐binding oligomerization domain (NOD)‐like receptor protein 3 (NLRP3) inflammasome activation and pore‐forming NLRP3‐caspase 1 (CASP1) substrate gasdermin D (GSDMD). In this form, MLKL can activate CASP1 inflammasome secretion before cell lysis.^[^ ^2^ ^]^ MLKL also plays a function in the pathogenesis of high in fat, fructose, and cholesterol (FFC) diet‐induced liver injury by inhibiting autophagy.^[^ ^3^ ^]^ Additionally, NETosis, another type of RCD characterized by the formation of neutrophil extracellular trap (NET), with necroptotic NETs specifically located on the membrane but whether they are surrounded by MLKL remains controversial.^[^ ^4^, ^5^ ^]^ MLKL expression and subsequent cell lysis are crucial factors determining the occurrence of RCD. Therefore, targeting MLKL provides new opportunities for therapeutic intervention in related diseases.^[^ ^6^ ^]^

The necroptosis driven by MLKL may provide protective effects or promote disease states under different circumstances.^[^ ^7^ ^]^ Regarding the relatively extensively studied tumor immunity, for example, in pancreatic ductal adenocarcinoma (PDAC), the elevated expression of MLKL recruits macrophages, enhances the “don't eat me” signal of tumor CD47, and induces the formation of extracellular traps (MET) in macrophages, ultimately supporting liver metastasis of PDAC.^[^ ^8^ ^]^ In hepatocellular carcinoma (HCC) patients, MLKL exhibits a negative correlation with the phosphorylation of AMPKα1, playing a key role in inhibiting autophagy and promoting hepatocarcinogenesis.^[^ ^9^, ^10^ ^]^ Besides, in HCC cells, MLKL deficiency increases cell susceptibility to parthanatos induced by metabolic stress in the liver microenvironment, thus enhancing anti‐tumor immune monitoring.^[^ ^11^ ^]^ In colorectal cancer (CRC) cells, MLKL supports basal autophagy, thereby providing protection against cell death. The inactivation of MLKL reduces this autophagy, creating a therapeutic vulnerability that could be exploited for CRC treatment.^[^ ^12^ ^]^ Therefore, inhibiting MLKL may exert anti‐tumor activity. In neurodegenerative diseases, such as Parkinson's disease^[^ ^13^, ^14^ ^]^ and Alzheimer's disease,^[^ ^15^ ^]^ inhibiting MLKL can also exert neuronal protective activity. The protective effect provided by MLKL is manifested in enhancing the antibacterial response and tumor suppression. For example, MLKL^−/−^ mice exhibit a greater bacterial burden and increased morbidity to systemic and localized Staphylococcus aureus and methicillin‐resistant S. aureus (MRSA) infections.^[^ ^4^, ^16^ ^]^ Membrane damage caused by necroptotic MLKL can prevent the progression of acute myeloid leukemia (AML) by triggering the activation of NLRP3 inflammasome.^[^ ^17^ ^]^ In addition to its direct killing effect, necroptotic signal transduction can also induce anti‐tumor immunity by activating cytokine transcription.^[^ ^18^, ^19^ ^]^ Beyond its role in the necroptosis pathways, MLKL has a broader range of non‐necroptotic functions, such as regulating gene expression and binding to lipids to block specific cellular processes.^[^ ^6^ ^]^ For example, MLKL‐knock‐out (KO) in pre‐adipocytes eliminates white adipocyte differentiation by strongly expressing Wnt10b (a ligand of the Wnt/β‐catenin pathway) and downregulating genes involved in fat metabolism, highlighting the therapeutic potential of targeting MLKL to inhibit obesity.^[^ ^20^ ^]^ In terms of lipid binding, phosphorylated MLKL (p‐MLKL) is capable of binding to phosphatidylinositol (4,5)‐diphosphate (PI(4,5)P2), thereby inhibiting the production of insulin‐stimulated PI (3,4,5) P3 in the plasma membrane (PM),^[^ ^21^ ^]^ which subsequently leads to the physiology of insulin resistance and the development of type 2 diabetes. In conclusion, MLKL is a promising therapeutic target for related diseases.

In recent years, there has been a dramatic increase in studies investigating MLKL activation, conformational change, dimerization, oligomerization, membrane translocation, membrane pore formation, and other related processes. These studies encompass the analysis of the crystal structures of mouse full‐length MLKL, the pseudokinase domain from human/mouse/rat/horse/pig, and N‐terminal 4HB domain. Furthermore, molecular dynamics (MD) simulations of MLKL in various oligomerization states have been modeled. However, the exact mechanism of MLKL activation remains elusive, and some reports on MLKL oligomerization are controversial. Even though the first report of MLKL inhibitor NSA has undergone more than 10 years of research, and there is still no clear conclusion on the exact mechanism of NSA's anti‐necroptosis effect. Moreover, although the non‐necroptotic functions of MLKL are extensive and seemingly unrelated, these functions appear to depend on the interactions between 4HB and other molecules. Consequently, summarizing the relevant research on MLKL protein structure is of paramount significance not only for understanding its necroptotic and non‐necroptotic functions, but also for exploring critical necroptosis‐related nodes and developing inhibitors.

## 2 Advances in Structural Biology of MLKL

### 2.1 Structures of MLKL

**Figure 1:**
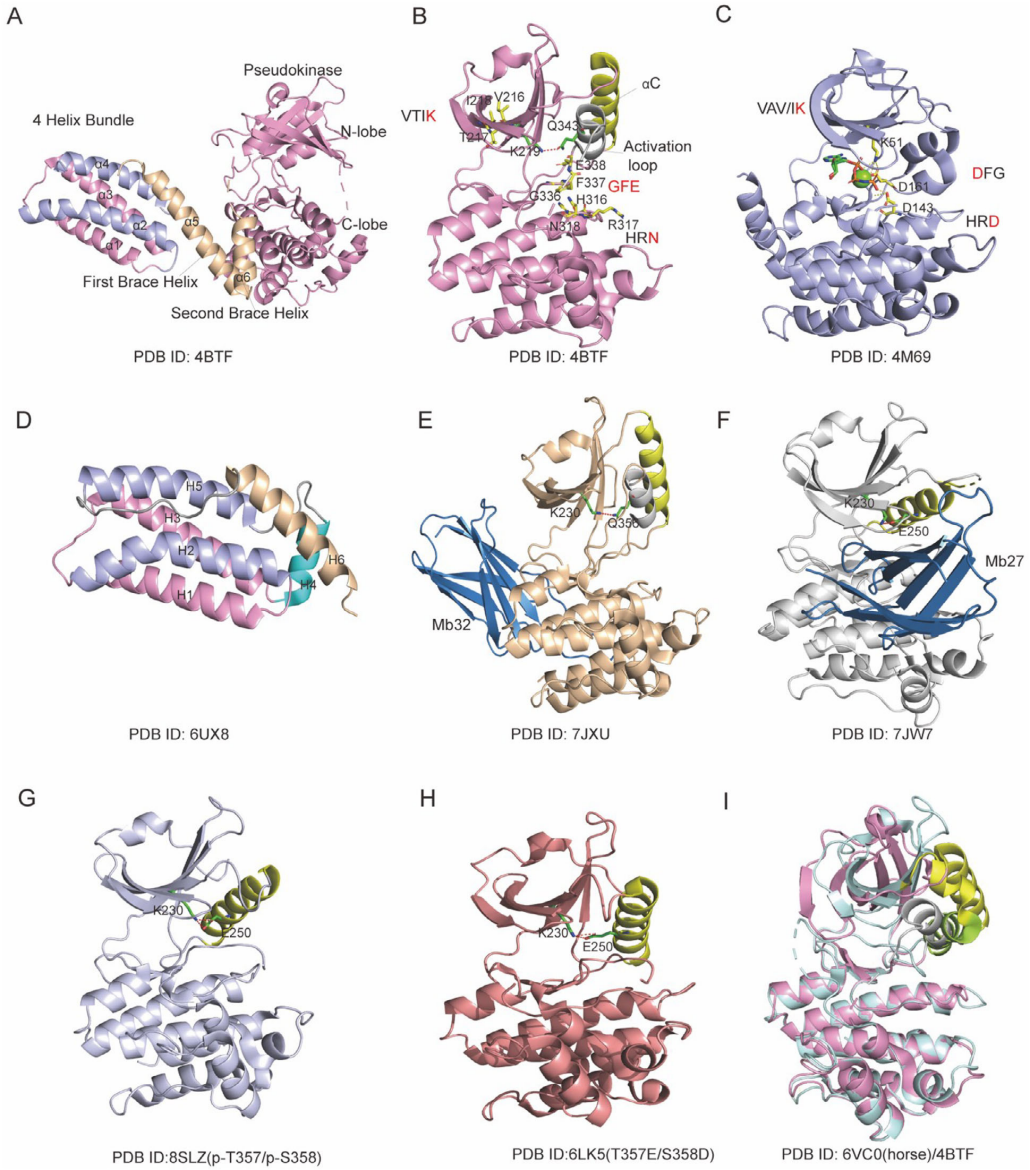
Structures of MLKL. A) The full‐length structure of mouse MLKL (PDB: 4BTF) reveals an N‐terminal helical bundle (α1‐α4), a two‐helix brace (α5‐α6), and a C‐terminal pseudokinase domain (N‐lobe and C‐lobe). B) In the mouse pseudokinase domain (PDB ID: 4BTF), the activation loop helix occupies the location where αC‐helix used to be, and is colored in gray and yellow, respectively. The pseudokinase domain absence of two out of the three conserved catalytic residues essential for phosphoryl transfer activity compared to RIPK3 (PDB ID: 4M69). VTIK, GFE, and HRN in mouse MLKL, VAV/IK, DFG, and HRD in RIPK3 C) or other traditional kinases. Additionally, this pseudokinase domain is in a closed‐inactive conformation, showing a hydrogen bond between K219 and Q343). D) The structure of human N‐terminal domain (PDB ID: 6UX8), including N‐terminal helical bundle (H1‐H5) and the first brace helix (H6). E) Human MLKL pseudokinase domain (PDB ID: 7JXU) in an open inactive conformation, showing a hydrogen bond between K230‐Q356. F) Human MLKL pseudokinase domain (PDB ID: 7JW7) in a closed‐active conformation, showing a salt bridge between K230 and E250. G,H) The crystal structures of phosphorylated (PDB ID: 8SLZ, lightblue) and phosphomimetic substituted hMLKL (PDB ID: 6LK5, salmon). I) Crystal comparison between horse MLKL pseudokinase domain (PDB ID: 6VC0, palecyan) and mouse MLKL pseudokinase domain (PDB ID: 4BTF, pink). Mouse activation loop (gray), mouse αC‐helix (yellow), horse αC‐helix (limon), and horse β3‐αC loop (yellow) are highlighted.

In 2013, the full‐length crystal structure of mouse MLKL was first characterized by Murphy et al.,^[^ ^22^ ^]^ However, the elucidation of the full‐length crystal structure of human MLKL remains an ongoing challenge. The Murphy's structure revealed that mouse MLKL consists of an N‐terminal four‐helix bundle (4HB, residues 1–117), a two‐helix linker (brace domain, residues 129–169), and a C‐terminal pseudokinase domain (residues 171–464) (**Figure** **1**A). MLKL is categorized as a “pseudokinase” due to the absence of two crucial catalytic residues out of the three that are typically conserved in protein kinases and are essential for phosphoryl transfer activity in its pseudokinase domain^[^ ^23^ ^]^ (Figure 1B). For example, in human RIPK3 (PDB ID: 4M69, Figure 1C), specific motifs facilitate specific interactions and functions, such as the Val‐Ala‐Ile‐Lys (VAIK) essential for ATP binding, the His‐Arg‐Asp (HRD) critical for catalytic activity, and the Asp‐Phe‐Gly (DFG) pivotal for Mg^2+^ coordination.^[^ ^24^ ^]^ In human MLKL (but not in other species), while the VAIK motif remains highly conserved as VTIK, the HRD motif is absent, appearing as HRN, and the DFG motif is typically replaced by GFE^[^ ^22^ ^]^ (Figure 1B). NMR and Crystallization efforts have successfully yielded the 4HB of human MLKL, revealing a slightly different conformation compared to that of mouse MLKL. Specifically, the sequence of human MLKL between α3 and α4 adopts an α‐helical structure (PDB ID: 6UX8, Figure 1D),^[^ ^25^ ^]^ whereas the corresponding α3‐ α4 loop in mouse MLKL (crystallization and NMR^[^ ^26^ ^]^) exhibits a disordered conformation. To clearly distinguish the two N‐terminal domains, for mouse MLKL, the four helices of 4HB are represented as α1‐α4, whereas the distinct first and second brace helices are labeled as α5 and α6, respectively. For human MLKL, H1‐H5 represent 4HB and H6 represents the first brace helix. After the activation loop of the pseudokinase domain is phosphorylated, specifically at T355/ S360/T357/S358 in human MLKL^[^ ^27^ ^]^ and Q343/S345 in mouse MLKL, a conformational change occurs. This structure transition propagates the activation signal through the brace helices to the 4HB domain. The brace domain determines the oligomerization and membrane translocation of MLKL and the release of cytotoxic 4HB.^[^ ^22^ ^]^

### 2.2 Different Activation Mechanisms of MLKL by RIPK3 Orthologues

In mice, phosphorylation of MLKL appears to be crucial for disrupting the hydrogen bond between K219 and Q343, which is required to maintain MLKL in a “closed‐inactive‐like” conformation (Figure 1B). Mutations in either of these two residues (K219M or Q343A) disrupts the binding and cause MLKL to exhibit an “open‐active” conformation, followed by oligomerization, and killing.^[^ ^22^ ^]^ This suggests that ATP is not required for activating mouse MLKL. In humans, MLKL has a “closed‐active” conformation (K230‐E250, Figure 1F) and a potential “open‐inactive” conformation characterized by an equivalent K230‐Q356 hydrogen bond (Figure 1E).^[^ ^28^ ^]^ Unlike mouse MLKL, the alanine substitution of K230 or Q356 is not sufficient to activate and elicit cell death.^[^ ^1^, ^28^ ^]^ Under basal conditions, human MLKL has been reported to bind to RIPK3.^[^ ^26^, ^28^, ^29^ ^]^ One possible scenario is that MLKL engaged by RIPK3 in this manner, locks the molecular switch to prevent MLKL activation, and alternatively, the pseudokinase domain of MLKL suppresses RIPK3.^[^ ^30^ ^]^ Following a necroptotic stimulus, MLKL dissociates from RIPK3 due to RIPK3‐mediated phosphorylation, which destabilizes the MLKL‐RIPK3 interaction,^[^ ^28^ ^]^ as evidenced by studies utilizing monobodies Mb27 and Mb32. Mb32 constitutively binds to the hinge region of inactive MLKL (K230‐ Q356, PDB ID: 7JXU, Figure 1E), while Mb27 binds to a site overlapping with the RIPK3 binding site on MLKL, which becomes accessible upon phosphorylation and dissociation from RIPK3 during necroptosis (PDB ID: 7JW7, Figure 1F).^[^ ^28^ ^]^ As a result of this dissociation, human MLKL acquires a “closed‐active” conformation with K230 bound to E250 via a salt bridge and forms an intact catalytic C‐spine (PDB ID: 7JW7, Figure 1F; PDB ID:8SLZ, Figure G). This, in turn, requires ATP or another metabolite ligand in the ATP‐binding site to modulate the MLKL conformational change.^[^ ^28^, ^31^ ^]^ The T357E/S358D mutation in human MLKL fosters the adoption of a closed conformation, reinforcing the concept that phosphorylation of human MLKL facilitates the transition from an open, inactive state to a closed, pro‐necroptotic state^[^ ^32^ ^]^ (PDB ID: 6LK5, Figure 1H). Therefore, the RIPK3 kinase domain: MLKL pseudokinase domain structure maintains the open conformation of the human MLKL pseudokinase domain.

The stable complexation between the RIPK3 kinase domain and MLKL pseudokinase domain is critical for the human RIPK3‐MLKL interaction. It has reported that the mouse RIPK3‐MLKL interaction and consequent phosphorylation adhere to a “kiss and run” kinase mechanism,^[^ ^33^ ^]^ because mutation at the phosphorylated site (K219M or Q343A) of activation loop of murine MLKL pseudokinase domain makes MLKL lethal, but the mutation of human MLKL pseudokinase domain (K230A or Q356A) does not affect the lethality of MLKL.^[^ ^1^, ^28^ ^]^ This difference in activation mechanism can be rationalized by the different conformations observed in the crystal structures of MLKL homologous genes, such as mouse, horse, and human MLKL pseudokinase domains.^[^ ^34^, ^35^, ^36^, ^37^, ^38^ ^]^ These conformations play a crucial role in the specific recognition of MLKL by RIPK3 in different orthologues, thus identifying significant variations in necroptosis signaling across different species.^[^ ^37^, ^39^ ^]^ More precisely, the role of activation loop phosphorylation in triggering MLKL activation appears to vary among homologous. Herein, we summarize the different activation mechanisms of MLKL orthologues with distinct pseudokinase domain conformations in vertebrates, as proposed by Davies et al.^[^ ^38^ ^]^ (**Table** **1**). Rat MLKL could not reconstitute the necroptotic signaling pathway in mouse MLKL^−/−^ MDF cells, despite both having a high similarity of 96% in their pseudokinase domain sequences. This may be since the crystal structure of mouse MLKL (K219‐ Q343, PDB ID: 4BTF) reveals it to be in an inactive state, while the crystal structure of rat MLKL shows it to be in an active state (K219‐E239, PDB ID: 6VBZ). However, phosphomimetic substitution in the rat MLKL pseudokinase domain (S345D) not only restored the necroptotic pathway in MLKL^−/‐^ MDF cells but also in human MLKL^−/−^ U937 cells, which appears to resemble the “kiss‐and‐run” activation mechanism of mouse MLKL. Pig MLKL can reconstitute the necroptotic pathways in both MLKL^−/−^ MDF and MLKL^−/−^ U937 cells. However, due to the lack of reported crystal structures of pig MLKL's pseudokinase domain, there is currently no research on the related activation mechanisms. Moreover, despite the relatively lower sequence conservation of MLKL pseudokinase domain between mice and horses compared to mice and rats, horse MLKL can reconstitute the necroptotic signaling pathway in mouse MLKL^−/−^ MDF cells, possibly because the activation loop of horse MLKL nestles within the pseudoactive site despite forms the salt bridge of K228‐E248 (PDB ID: 6VC0), thus remaining in an inactive state, similar to mouse MLKL. Besides, the spatial arrangement of the β3‐αC helix in horse MLKL (PDB ID: 6VC0) occupies the position of the αC helix of mouse MLKL (PDB ID: 4BTF). This structural similarity facilitates the recognition and binding of RIPK3 in mice (Figure 1I). The alanine‐mutated horse MLKL activation loop (T356A/S357A) cannot reconstitute the necroptotic pathway in MLKL^−/−^ MDF cells, indicating that the possible activation mechanism of horse MLKL is similar to that of mouse MLKL. However, the phosphomimetic substituted horse MLKL activation loop (T356E/S357E) also fails to reconstitute the necroptotic pathway in MLKL^−/−^ MDF cells, suggesting that the possible activation mechanism of horse MLKL resembles that of human MLKL.^[^ ^38^ ^]^ In a word, although the activation mechanisms of MLKL in these vertebrates are not fully understood, it is undeniable that they are related to the conformational changes of the MLKL activation loop. Finally, it is necessary to emphasize the evolutionary significance of the divergence in MLKL activation. The murine “kiss‐and‐run” mechanism may enable rapid immune responses against intracellular pathogens. Studies demonstrate that the high basal expression levels of RIPK3 in murine immune organs and gastrointestinal tract facilitate RIPK3‐MLKL pathway activation, contributing to non‐immune cell‐mediated host defense against Listeria invasion.^[^ ^40^ ^]^ In contrast, the evolution of stable RIPK3‐MLKL complexes in humans appears to favor controlled immunogenicity and prevention of excessive inflammation.^[^ ^41^ ^]^ This is supported by the following evidence. Studies have shown that MLKL has three key characteristics of host defense factors: 1) regulated by immune signals,^[^ ^42^ ^]^ 2) having viral homologues/viralological features,^[^ ^43^ ^]^ 3) evolving rapidly in bats and primates.^[^ ^41^ ^]^ All these indicate that the evolution of human MLKL is aimed at achieving balanced defense functions.

**Table 1:** Summary table of different activation mechanisms of MLKL homolog proteins with different pseudokinase domain conformations.

Species (MLKL orthologues)	The activation loop state in the MLKL pseudokinase domain crystal	Mouse		Human		The reasons for the success or failure of the reconstruction of necroptotic signaling
		Pseudokinase domain identity (similarity) %	MLKL^–/–^ MDF + orthologues reconstitute necroptotic signaling	Pseudokinase domain identity (similarity) %	MLKL^–/–^ U937 + orthologues reconstitute necroptotic signaling	
Mouse	Inactive (K219‐Q343, PDB ID: 4BTF)	__	√	69.3 (86.6)	×	__
Mouse (S345D)	/	/	√	/	/	the mouse RIPK3‐MLKL interaction and phosphomimetic substitution adhere to a “kiss and run” kinase mechanism
Human	Active (K230‐E250, PDB ID:4MWI)	69.3 (86.6)	×	__	√	__
Human (T357E/S358E)	/	/	/	/	×	Phosphomimetic substitution of human MLKL T357/S358 leads to the inability of RIPK3: MLKL complex, which is the key for human MLKL activation.
Pig	No crystals	66.1 (87.3)	√	68.3 (91.8)	√	NA
Rat	Active (K219‐E239, PDB ID: 6VBZ)	87.9 (96.2)	×	68.1 (87.9)	×	The inability of rat MLKL to reconstitute the mouse necroptosis pathway could be attributed to the respective active kinase‐like and inactive kinase‐like conformations of the rat and mouse MLKL pseudokinase domains.
Rat (S345D)	/	/	√	/	√	Phosphomimetic substitution in the rat MLKL pseudokinase domain can trigger necroptosis, like that of mouse MLKL.
Horse	Inactive (K228‐E248, the activation loop nestles within the pseudoactive site, PDB ID:6VC0)	67.6 (87.1)	√	70.2 (89.0)	×	The capacity of horse MLKL to reconstitute the mouse necroptosis pathway due to: 1. The additional helix N‐terminal to the αC helix in horse MLKL, which spatially occupies the position of the mouse activation loop helix. 2. The activation loop of horse MLKL also adopts an inactive‐like conformation.
Horse (T356A/S357A)	/	/	×	/	×	The horse MLKL T356A/S357A mutation leads to the inability of reconstitute the mouse necroptosis pathway just like mouse MLKL mutation
Horse (T356E/S357E)	/	/	×	/	×	The phosphomimetic substitution of horse MLKL T356/S357 leads to the inability of reconstitute the mouse necroptosis pathway just like human MLKL mutation

NA: Not Applicable.

The MD simulations by Garcia et al. showed that S345 in the activation loop is oriented toward the neighboring helix and residue T235 in the wild‐type mouse MLKL. However, upon phosphorylation of S345, the phosphate group relocates to interact with the solvent, leading to a disruption in both the structure and alignment of the activation loop.^[^ ^28^ ^]^ Then, the phosphorylation event of the pseudokinase domain is transmitted to the 4HB domain through the brace helix, triggering a conformational change in the 4HB domain.^[^ ^1^, ^44^, ^45^ ^]^ Davies et al. revealed through a rosettaremodel modeling analysis that the phosphorylated activation loop of horse MLKL transitions from the pseudoactive site to an extended conformation, causing the disruption of the Q355‐C284 interaction. These findings indicate that the introduction of phosphate groups destabilizes the concealed activation loop structure, which may inhibit the occupation of the pseudoactive site and enhance its flexibility to influence the association and dissociation of RIPK3.^[^ ^38^ ^]^ The exact function of RIPK3‐mediated phosphorylation of the MLKL pseudokinase domain activation loop in human MLKL activation remains elusive, primarily due to the ambiguity surrounding the specific residues within the activation loop that are prone to be affected by phosphorylation. Consequently, conducting MD simulations to investigate this concept poses significant challenges, hindered by the current structural limitations.^[^ ^26^, ^38^ ^]^

Other studies have shown that the activation of mouse MLKL requires not only RIPK3‐mediated phosphorylation of the activation loop, but also requires K219 to bind the Ubiquitin (Ub) signal chain linked to K63 during the necroptotic process.^[^ ^45^ ^]^ Although RIPK3‐mediated phosphorylation of S345 has been reported to disrupt the hydrogen bond between K219 and Q343, this phosphorylation event at the S345 site consistently occurs in tandem with ε‐amino ubiquitination of K219. MD simulations of K219ub S345phos MLKL suggested that the attachment of Ub to K219 appears to impact the flexibility of the 4HB domain in phosphorylated MLKL, potentially playing a crucial role in MLKL polymerization. This clarification explains why ubiquitination specifically targets the active, phosphorylated form of MLKL. Furthermore, it introduces the intriguing possibility that ubiquitination of K219 serves to stabilize the activated conformation of mouse MLKL, thereby enhancing its contribution to necroptosis.^[^ ^45^ ^]^

### 2.3 Dynamics Studies of Human MLKL

**Figure 2:**
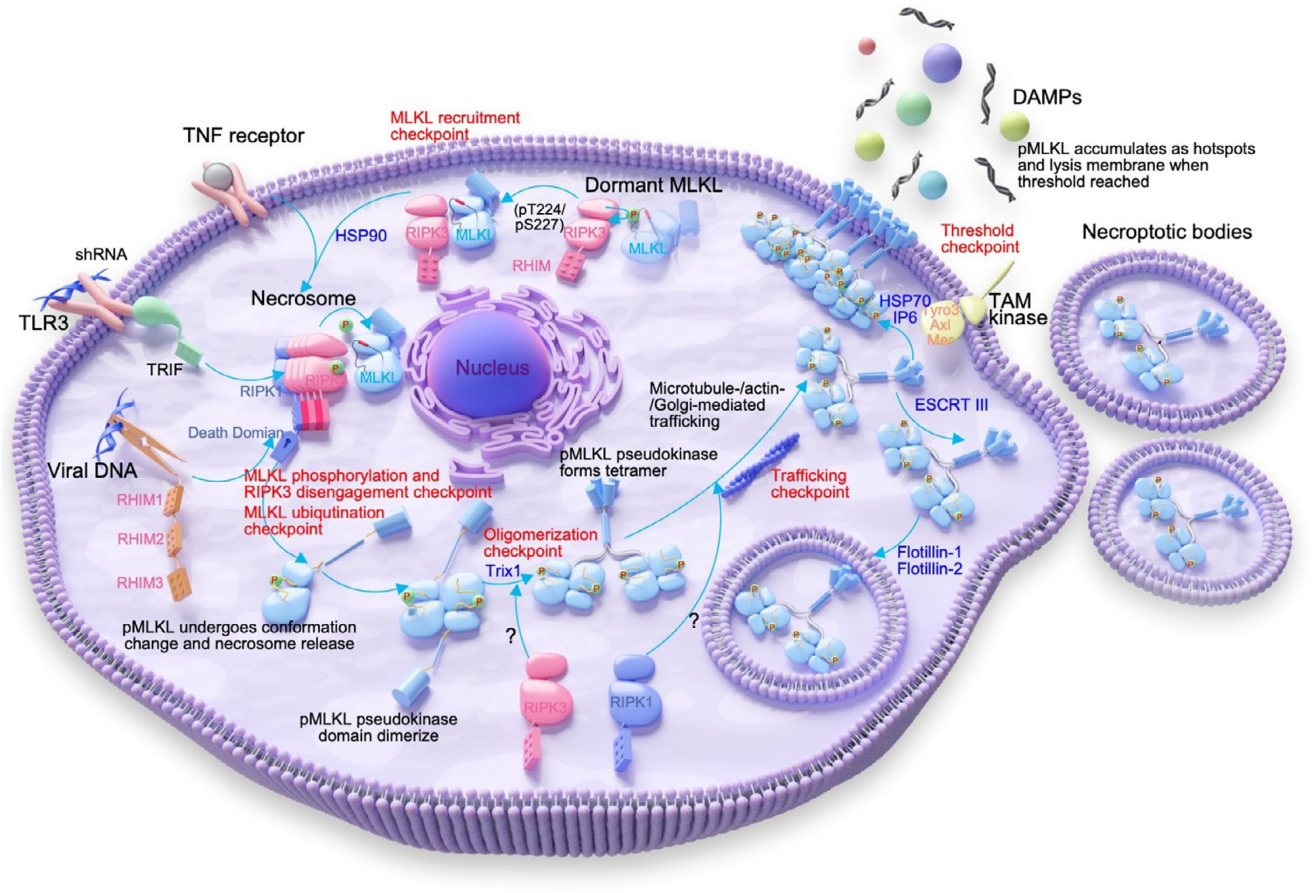
A summary of the dynamics of human MLKL activation and important checkpoints in typical necroptotic signaling pathways. Necroptosis can be triggered by various receptors including TNF‐receptor, Toll‐like receptors (TLRs), Z‐DNA‐binding protein 1 (ZBP1)/DAI), upon interaction with their specific ligands. Under basic conditions, RIPK3 forms a stable complex with MLKL through the phosphorylation of T224 and S227, in which both RIPK3 and MLKL are inactive (MLKL recruitment checkpoint). Then, RIPK3 recruits MLKL into the necrosome, where RIPK1 and RIPK3 form oligomers through a RHIM‐mediated amyloid fibril formation, leading to the activation of RIPK3 through autophosphorylation. This process subsequently recruits MLKL and activates it through RIPK3‐mediated phosphorylation (MLKL phosphorylation and RIPK3 disengagement checkpoint). Meanwhile, mouse MLKL K219 was reported to occur ubiquitination checkpoint. The ubiquitination process of the K230 protein in human MLKL may exist, but there are no relevant reports. Once phosphorylated, MLKL undergoes a conformational change, exposing the killer 4HB domain and activating its executioner function. Human MLKL then undergoes dimerization, oligomerization (Oligomerization checkpoint), and transport to the plasma membrane (Trafficking checkpoint) via the actin, Golgi, and microtubule systems, where it accumulates into higher order hotspots (Threshold checkpoint), leading to the formation of membrane pores and the release of DAMPs. Six checkpoints are marked in red, and various positive and negative regulatory proteins are marked in black. Abbreviations: RHIM, RIP homotypic interaction motif; TLR, Toll‐like receptor; TNF, tumor necrosis factor; ZBP, Z‐DNA‐binding protein 1. ESCRT‐III, endosomal sorting complex required for transport; Trx1, thiol oxidoreductase thioredoxin‐1; DAMPs, damage‐associated molecular patterns.

Although the specific molecular mechanisms underlying the activation of MLKL vary across different species, and this activation appears to be a continuous process, there are some common checkpoints that can divide this process into several distinct stages. Here, we mainly focus on human MLKL to summarize its role and behavior in these stages (**Figure** **2**). The initial stage involves the stable recruitment of MLKL to the necrosome.^[^ ^46^ ^]^ The RIP homotypic interaction motif (RHIM)‐containing proteins such as RIPK1, RIPK3, TIR‐domain‐containing adapter‐inducing interferon‐β (TRIF), and Z‐DNA binding protein 1 (ZBP1) are indispensable for the formation of the necrosome. Notably, recent studies have revealed a fundamental species‐specific difference in necroptosis execution: human RIPK1 is essential for ZBP1‐mediated necroptosis, whereas murine RIPK1 paradoxically inhibits ZBP1‐triggered necroptosis – representing a critical divergence in necroptotic pathways between humans and mice.^[^ ^47^, ^48^ ^]^ During the recruitment process, a pre‐existing dormant complex of RIPK3 and MLKL (in an open, inactive conformation, involving residues K230‐Q356) translocate from the cytoplasm to the necrosome. Han et al. used super‐resolution microscopy to visualize necrosome and found that only RIPK3 oligomers with sizes of tetramer or above allow MLKL, recruited by phosphorylated RIPK3, to oligomerize for necroptosis.^[^ ^49^ ^]^ Once entering the necrosome, MLKL undergoes further activation and subsequently forms larger clusters formation in the perinuclear region of cells.^[^ ^29^ ^]^ The above interaction between RIPK3 and MLKL is facilitated by their individual kinase and pseudokinase domains^[^ ^50^ ^]^ and can only be biophysically detected when using human recombinant proteins (e.g., PDB ID: 4M69, mouse RIPK3 kinase ‐MLKL pseudokinase domain form complex), rather than in vivo detection.^[^ ^22^, ^34^ ^]^ In the subsequent checkpoint, RIPK3 phosphorylates the pseudokinase domain of MLKL, inducing a structural change that leads to the detachment of MLKL from the necrosome and exposure of MLKL's 4HB executioner domain.^[^ ^28^, ^51^ ^]^ The recent identification of mouse MLKL K219 (maybe K230 in human MLKL) ubiquitination after MLKL phosphorylation has also highlighted a novel checkpoint in the regulation of MLKL activation.^[^ ^45^, ^52^ ^]^ After detaching from RIPK3, phosphorylated MLKL forms a head‐to‐tail, back‐to‐back dimer structure in pseudokinase domain.^[^ ^27^ ^]^ A species‐divergent model implied that MLKL dimerization is a conserved step in the activation of MLKL in rat and horse, rather than in mice.^[^ ^32^ ^]^ However, in mice, the dimerized activation step can be defined as the formation of the RIPK3‐MLKL complex.^[^ ^32^ ^]^ Further research has shown that when MLKL is activated through enforced dimerization, it is unable to induce cell death in the absence of RIPK1.^[^ ^53^ ^]^ Similar to RIPK1, RIPK3 also likely plays some role downstream of MLKL phosphorylation, as the recruitment of MLKL to the necrosome may be essential for its interaction with downstream regulators, such as trafficking partners ^[^ ^35^ ^]^ or chaperones.^[^ ^25^, ^54^ ^]^ The fourth checkpoint is oligomerization (tetramerization) of full‐length human MLKL, triggered by phosphorylation‐dependent dimerization of the pseudokinase domain.^[^ ^27^ ^]^ The precise stoichiometry of human MLKL oligomers has been a subject of continuous discussion. Recent research increasingly indicated that conformational changes in the pseudokinase domain drived tetramerization of human MLKL.^[^ ^27^, ^34^ ^]^ As an oligomer, MLKL is transported to PM, representing a pivotal trafficking checkpoint in necroptosis. This process in human MLKL relies on intricate interactions with the actin, Golgi, and microtubule systems.^[^ ^35^ ^]^ Finally, in both human and murine cells, the accumulation of MLKL at the PM serves as a checkpoint to control membrane rupture. Here, large, micron‐sized clusters form, where the 4HB domains of MLKL units cooperate to disrupt the membrane integrity, resulting in cell swelling and eventual release of cellular contents into the extracellular environment ^[^ ^35^ ^]^ (Figure 2).

Among these regulatory factors, some positively modulating targets such as inositol phosphates (IPs), HSP90, HSP70, and TAM (Tyro3, Axl, and Mer) kinase play pivotal roles (Figure 2). Notably, highly phosphorylated inositol species, such as IP6, can directly bind to the N‐terminal executive domain (NED) of MLKL. Through their electronegative charges, these IPs can induce important structural rearrangement within the NED, ultimately disrupting the self‐inhibitory region (H6 in human MLKL and α5 in mouse MLKL), thereby unleashing MLKL's activity.^[^ ^55^, ^56^, ^57^, ^58^ ^]^ HSP90 plays a pivotal role in maintaining the stability of MLKL, disrupting HSP90 function effectively prevents necrosome formation and significantly diminishes MLKL phosphorylation. Furthermore, HSP90 co‐expression enhances MLKL oligomerization and membrane translocation.^[^ ^54^, ^59^, ^60^ ^]^ HSP70, another chaperone protein, contributes to MLKL regulation through its substrate binding domain (SBD), which interacts with the NED of MLKL to modulate the polymerization step after tetramerization.^[^ ^61^ ^]^ On the PM, Tyr376 of human MLKL can be phosphorylated by membrane‐associated TAM kinases. This phosphorylation event promotes further oligomerization of membrane‐ binding MLKL and facilitates necroptosis execution.^[^ ^62^ ^]^ In conclusion, the activation of MLKL requires not only the phosphorylation of MLKL by RIPK3, but also positive regulation of these in necroptotic pathways. Of course, the activation of MLKL is not always dependent on RIPK3. For instance, Zhan et al. demonstrated that CAMK2/CaMKII (calcium/calmodulin dependent protein kinase II) could activate MLKL during short‐term starvation and promotes autophagic flux.^[^ ^63^ ^]^ In addition to positive regulatory proteins, MLKL also modulated by negative regulatory proteins, such as thiol oxidoreductase thioredoxin‐1 (Trx1) and endosomal sorting complex required for transport (ESCRT‐III). Trx1 plays a crucial role in maintaining cellular redox balance by catalyzing thiol disulfide exchange reactions to reduce disulfide bonds on specific protein targets. Trx1 has been found to prevent necroptosis by impeding the formation of disulfide bonds and polymerization of MLKL.^[^ ^64^ ^]^ ESCRT‐III emerges as a key downstream negative regulatory target of MLKL, playing a pivotal role in alleviating the detrimental effects of excessive MLKL accumulation on PM, thereby preserving membrane integrity^[^ ^65^ ^]^ and enabling the generation of crucial cytokines, damage‐associated molecular patterns (DAMPs), and an inflammatory reaction^[^ ^66^ ^]^ (Figure 2). In addition, Zhu et al., and Garnish et al., have successively reported that MLKL undergoes inhibitory phosphorylation (human MLKL S83 and mouse MLKL S82) during the process of necroptosis activation.^[^ ^67^, ^68^ ^]^ Among them, the phosphorylation of mouse MLKL S82 has been proved to be essential for the normal development of mice and can prevent inflammatory damage and neonatal death caused by necroptosis.^[^ ^69^ ^]^ However, the kinase that leads to this inhibitory phosphorylation has not been reported yet.

#### 2.3.1 RIPK3‐MLKL Complex Formation

**Figure 3:**
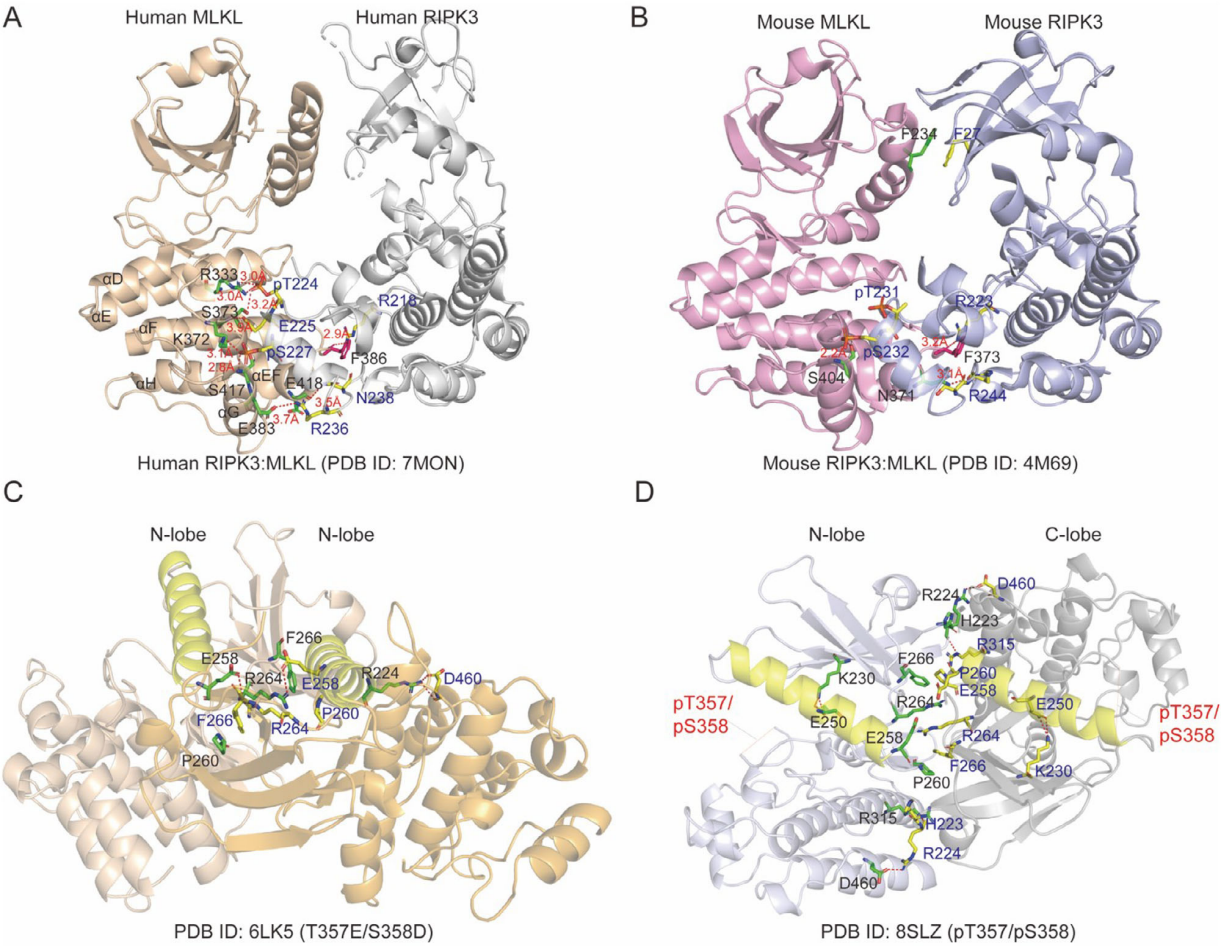
Crystal structures of RIPK3: MLKL complexes and MLKL pseudokinase domain dimers. A) Orthogonal view of the co‐crystal structure of human RIPK3 (residues 1–316; C3S, C110A, colored gray) and human MLKL (residues 190–471, colored wheat) (PDB ID: 7MON). The salt bridges between the C‐lobes of human RIPK3 and MLKL, including human MLKL K372:RIPK3 E225, MLKL K372:RIPK3 pS227, and MLKL E418:RIPK3 R236, combined with the MLKL R333:RIPK3 pT224 interaction, create a unique electrostatic landscape within the C‐lobes of the human RIPK3: MLKL complex. Side chains engaged in electrostatic interactions are represented as sticks, with these interactions highlighted by red dashed lines. αD‐αH are highlighted in red. B) Orthogonal view of the co‐crystal structure of mouse RIPK3 (colored light blue) and mouse MLKL (colored pink) (PDB ID: 4M69). The C‐lobe electrostatic and hydrophobic interactions within the mouse RIPK3: MLKL structure are represented as sticks, with these interactions highlighted by red dashed lines. C) The symmetric crystal structure of the MLKL kinase‐like domain phosphomimic mutants (T357E/S358D, PDB ID: 6LK5). The αC‐helix in N‐lobe is highlighted in yellow, the key residues in MLKL dimer interface such as R224, E258 are represented as sticks, with some interactions highlighted by red dashed lines. D) The crystal structure of the phosphorylated human MLKL pseudokinase domain dimer. The p‐MLKL pseudokinase domain is in a closed conformation (PDB: 8SLZ) and exhibits typical active kinase characteristics, including a salt bridge between β3‐Lys (VAIK230) and αC‐Glu (E250). The αC‐helix in N‐lobe is highlighted in yellow, the key residues in MLKL N‐lobe such as R224, E258, K230, E250 are represented as sticks, with interaction highlighted by red dashed lines.

Recruitment of MLKL by RIPK3 plays an important role in necroptotic execution, mutations occurring at the interaction interface of RIPK3 – MLKL dormant complex, or competitive binding of viral MLKL orthologs to RIPK3, have the capability to inhibit necroptotic signaling in human and murine cells.^[^ ^34^, ^37^, ^43^ ^]^ Through phosphoproteomics and mutational analyses, pT224 and pS227 in human RIPK3 (T231/S232 in mouse RIPK3^[^ ^37^, ^50^ ^]^) were determined to be essential for synergistically facilitating a stable interaction with MLKL, thereby promoting necrosome formation.^[^ ^1^ ^]^ However, the phosphorylation of RIPK3 at T224 and S227 is not necessary for MLKL phosphorylation, as both the single and double mutants retained the ability to phosphorylate MLKL but did not initiate necroptosis.^[^ ^1^, ^37^, ^46^ ^]^ Previous studies have shown that the spatial arrangement of the paired phosphate sites is a key determinant factor for MLKL phosphorylation.^[^ ^29^, ^50^ ^]^ The RIPK3‐ MLKL interface mutants (V220E, L222D, and A232R) also eliminated cell death without affecting MLKL phosphorylation.^[^ ^29^ ^]^ These findings suggest that while the phosphorylation of MLKL by RIPK3 is a prerequisite for necroptosis, it alone is insufficient to fully orchestrate and execute this RCD pathway. Instead, the key factor for mediating necroptosis seems to be the effective recruitment of MLKL to RIPK3 or the necrosome in a stable manner.^[^ ^46^ ^]^ In addition, T224 of human RIPK3 is conserved only in the horse and pig orthologs, not in the murine counterpart (**Figure** **3**A,B), which likely plays a role in the species‐specificity of the RIPK3‐ MLKL interaction. Hence, necroptosis in human cells can be reconstituted with pig MLKL, while mouse MLKL cannot.^[^ ^38^ ^]^ Furthermore, a stable complex between recombinant RIPK3 and MLKL is formed only in a co‐expressed situation. Notably, phosphorylation of T224 and S227 is significantly elevated in the RIPK3‐ MLKL complex compared to independently expressed RIPK3 kinase domain.^[^ ^29^ ^]^ These findings indicate that autophosphorylation of RIPK3 at T224 and S227 is a crucial prerequisite not only for the formation of a stable complex with MLKL, but also unexpectedly, depends on the presence of MLKL.^[^ ^29^, ^46^ ^]^ Of course, it is necessary to point out here that in other forms of RIPK3‐mediated necroptosis, MLKL is not always indispensable. In some studies, the knockdown or knockout of MLKL does not always reduce cell death or tissue damage. In certain contexts, RIPK3 can activate CaMKII and other downstream pathways independently of MLKL, particularly in the setting of inflammation and sepsis.^[^ ^70^, ^71^ ^]^

Although with distinct characteristics, the αC helix serves as the central point of N‐lobe interface interaction in both human and mouse RIPK3‐MLKL complexes. The elongated αC helix of mouse MLKL, compared to its apo form, exposes F234 for *π*–*π* interactions with F27 in RIPK3 αC helix hydrophobic interface, which has been validated as crucial for mouse MLKL – RIPK3 binding^[^ ^50^ ^]^ (Figure 3B). Therefore, the absence of these phenylalanines in human RIPK3 and MLKL likely contributes, in part, to their inability to interact with their mouse counterparts.^[^ ^29^ ^]^ In mice, the salt bridges observed at the C‐lobe interface in human RIPK3‐ MLKL complex are absent, suggesting that this distinct electrostatic landscape in humans may explain the species‐specific recognition mechanisms between RIPK3 and MLKL.^[^ ^29^ ^]^ Moreover, the reduced number of salt bridges present appears to correlate with the observed decreased stability of mouse RIPK3‐ MLKL complex compared with the human counterpart (Figure 3A,B). Additionally, the importance of the hydrophobic core of the C‐lobe interface has also been emphasized. The conserved Phe/Tyr (located in the αEF‐αF loop) in MLKL C‐lobe such as humans (F386),^[^ ^34^ ^]^ mice (F373),^[^ ^38^, ^50^ ^]^ horses (Y385),^[^ ^38^ ^]^ and viruses (F202)^[^ ^31^ ^]^ are essential for the interaction of the C‐lobe of the corresponding RIPK3. Earlier biochemical research suggested that the RIPK3 αG helix plays a significant role in determining the species specificity of MLKL.^[^ ^37^ ^]^ In conclusion, the αC‐helix of N‐lobe and C‐lobe of MLKL pseudokinase domain are associated with recognition binding and species selectivity of RIPK3.

#### 2.3.2 MLKL Dimerization

Pseudokinase domain is reported to function as a molecular switch, in which MLKL pseudokinase domain activation loop phosphorylated by RIPK3 triggers a conformational change, releasing an autoinhibitory protein–protein interaction with the executioner 4HB domain.^[^ ^22^, ^51^ ^]^ pseudokinase domain dimerization is triggered by RIPK3‐mediated phosphorylation, which is a crucial step in inducing oligomerization of full‐length proteins.^[^ ^27^, ^32^ ^]^ Currently, researchers have proposed two molecular mechanisms for pseudokinase domain dimerization. In the first study, the researchers investigated the potential mechanism of dimerization by crystallizing recombinant proteins of the MLKL pseudokinase domain in phosphor‐site mutants (T357A/S358A, PDB: 6LK6) and phosphomimic mutants (T357E/S358D, PDB ID: 6LK5). They found that only one crystallographic dimer had symmetric adjacent N‐termini (Figure 3C) and demonstrated that MLKL dimerization did not impede its interaction with RIPK3. Additionally, the dimeric form of the mouse MLKL pseudokinase domain conflicted with the monomeric full‐length structure of mouse MLKL (PDB ID: 4BTF, autoinhibited), suggesting that dimerization of the pseudokinase domain discourages the autoinhibitory conformation. Besides, this dimer formation mechanism was proposed for another reason, which is the mutations in the MLKL dimer interface (PDB ID: 6LK5) (H223A/R224A/E258A/P260G/R264A/F266A, AAAGAA) block the transition from monomer to oligomer. (Figure 3C). However, mutations in four key auto‐oligomerized alanine residues (L162A/L165A/M169A/I172A, 4A) within the brace region are reported can only prevent the formation of oligomers, confirming that this pseudokinase domain dimerization was the initial step.^[^ ^32^ ^]^

Based on the crystal structure of the phosphorylated human MLKL pseudokinase domain (PDB ID: 8SLZ) and biophysically verification, the second dimerization mechanism was proposed, in which MLKL forms a head‐to‐tail, back‐to‐back dimer structure^[^ ^27^ ^]^ (Figure 3D). In this dimer, the brace region formed a continuous long helix extending ≈80Å, instead of the two shorter helices observed in full length mouse MLKL structure (PDB ID: 4BTF). The binding site of RIPK3 was occupied by Mb27, indicating that MLKL dimerization separates from RIPK3, and the closed conformation is coupled to the dimerization of the human MLKL pseudokinase domain (Figure 3D). This dimerization also has similar hydrophobic interactions (H223, R224, E258, P260, R264, F266, R315, D460).^[^ ^27^ ^]^

#### 2.3.3 MLKL Tetramerization

RIPK3‐mediated MLKL phosphorylation releases brace helices,^[^ ^28^, ^34^, ^51^ ^]^ thereby inducing the formation of MLKL oligomers.^[^ ^22^, ^72^, ^73^, ^74^, ^75^ ^]^ The perturbation of the brace region is conducive to transforming MLKL's autoinhibited conformation into active oligomer conformation. For example, in mice, homozygosity for MLKL^S131P^ formed high molecular weight membrane‐associated complexes in the absence of necroptotic stimulation,^[^ ^68^ ^]^ and MLKL^D139V^ caused fatal perinatal inflammatory syndrome and hematopoietic defects.^[^ ^68^ ^]^ The first brace helix and neighboring loop are reported as crucial components of the molecular switch mechanism that transmit pseudokinase domain phosphorylation to activate the killing function of the 4HB domain. The first brace helix is also involved in MLKL membrane translocation.^[^ ^76^ ^]^ Davies et al. proposed that the residues within the second brace helix serve as the interface for MLKL oligomerization (161EINKTLKQ168) by Small Angle X‐ray Scattering (SAXS) and SASREF modeling, facilitating trimerization of the mouse MLKL NED.^[^ ^33^ ^]^ So far, there have been many reports on the order of magnitude of MLKL oligomers, but these reports seem unable to deny the fact that it has been reported that MLKL in necrosome^[^ ^35^, ^49^ ^]^ and hotspots^[^ ^35^ ^]^ is giant. Herein, we summarize the relevant research on the tetramerization and high‐order polymerization of human MLKL.

Meng et al. supported the concept of MLKL tetramers through negative staining electron microscopy and modeling, proposing that MLKL pseudokinase domain dimerization first, resulting in the brace helices extension into a single helix, which in turn promoted MLKL assembly into pro‐necroptotic tetramers.^[^ ^27^ ^]^ In their model, the brace helices from the four prepolymers were assembled into a coil that was primarily crimped by the hydrophobic side chain core (F148, L151, I158, L162, L165, M169, I172, L176). Mutants of these hydrophobic residues could eliminate MLKL oligomerization and necroptosis.^[^ ^27^ ^]^ Mass spectrometry analysis showed that the MLKL tetramer co‐expressed with RIPK3 was linked by disulfide bonds via C184. However, human MLKL C184S led to oligomerization defects but did not inhibit necroptosis.^[^ ^27^ ^]^ Thus, the disulfide form of activated MLKL may be an artifacts arising from its proximity within hot spots or the necrosome. While these forms could serve as potential diagnostic markers for MLKL activation, the process may not be directly related to its functional activity.

**Figure 4:**
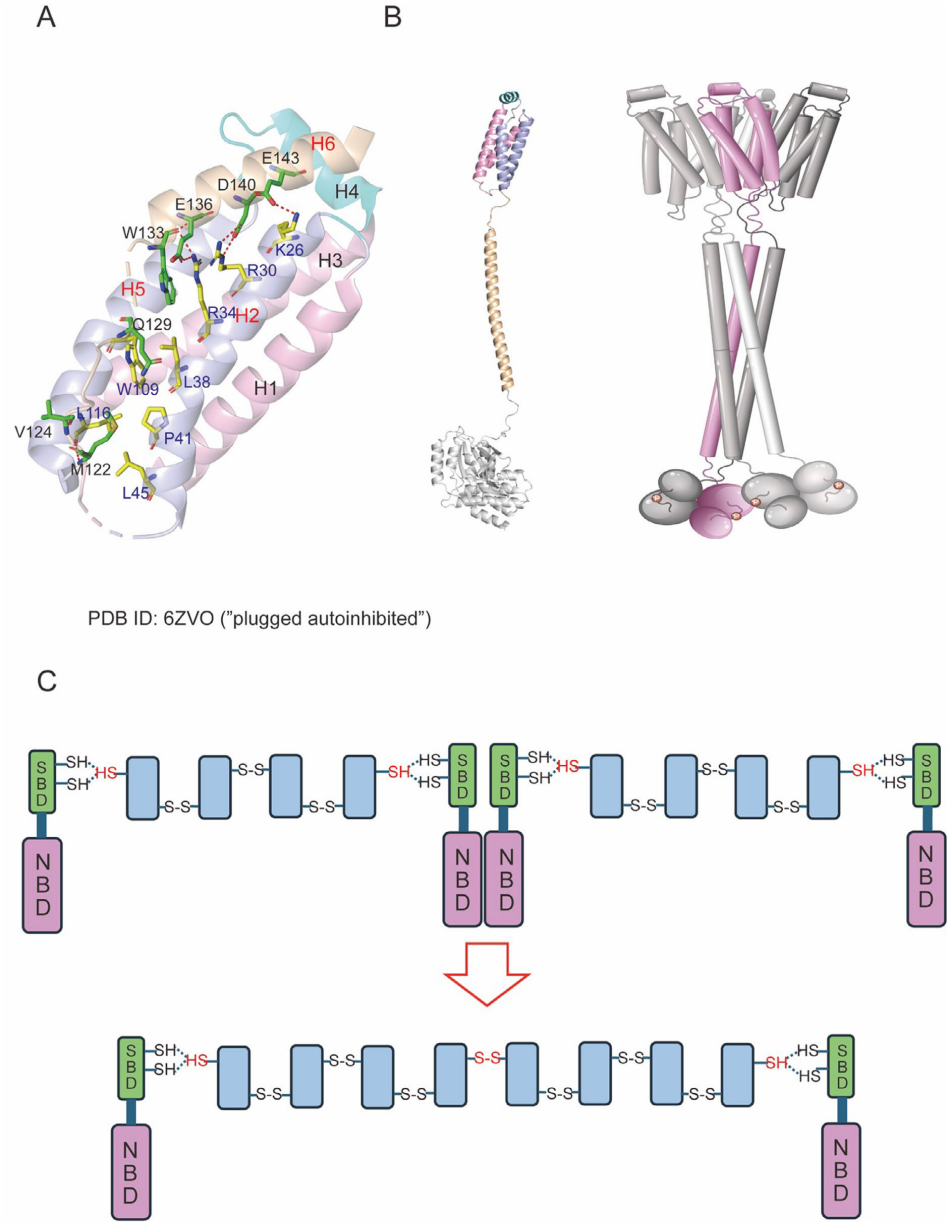
The oligmerization of full‐length human MLKL. A) The H2 and H5 helices of the 4HB domain mediates the interaction with the brace helix (H6, wheat). The key residues in MLKL N‐lobe such as R224, E258, K230, E250 are represented as sticks, with interaction highlighted by red dashed lines. B) The model of human MLKL tetramer.^[^ ^27^ ^]^ Pseudokinase domain dimer is within p‐MLKL tetramer. C) Proposed mechanism of the MLKL octamer formation from Johnston et al.^[^ ^55^ ^]^ HSP70 binds to the N‐terminal domain of the MLKL tetramer, employing cysteines 574 and 603 to shield the functional cysteines within the tetramer. This protective action facilitates the correct formation of new disulfide bonds (red line) between tetramers, thereby promoting polymerization.

MLKL tetramerization also requires 4HB to be released by the extended autoinhibited brace helix. In the inactive mouse MLKL structure (PDB ID: 4BTF), a series of electrostatic interactions (R34‐E135, R30‐D139, and K26‐E142) were observed between α2 helix of the 4HB domain and the autoinhibited α5 helix of the brace region.^[^ ^22^ ^]^ In human MLKL (PDB ID: 6ZVO), a similar interaction also causes the autoinhibited helix H6 to occupy the H2‐H5 helix groove, thus limiting the release and lethality of 4HB (**Figure** **4**A). These observations led to a plausible “plug‐release” mechanism for the brace helix that limited the executioner function of MLKL.^[^ ^77^, ^78^ ^]^ In Meng's human MLKL tetramer model, the 4HB domain releasing itself from the autoinhibited helix H6, leaving a groove between H2 and H5, specifically, the H5 helix of one 4HB domain inserts into the groove located between the H2′ and H5′ helices of the adjacent protomer (Figure 4B). This interaction is mediated by salt bridges formed between H5 (D100, D107) and H2′ (K26, R30, R34), as well as hydrophobic interactions between H5 (L114, V118) and C‐terminal ends of H2′ (L38, P41, L45) and H5′ (W109, L116).^[^ ^27^ ^]^ The previous report has confirmed that disruption of the H2 R34‐H5' D107 salt bridge could block necroptosis.^[^ ^25^, ^34^, ^78^ ^]^ In their model, the 4HB tetramer creates a sizable positively charged central pocket, which could interact with the negatively charged headgroups of phosphoinositols known to bind to MLKL,^[^ ^26^, ^34^, ^73^, ^78^ ^]^ concluding that the 4HB domain recombination in human MLKL tetramer is a prerequisite for PM binding.

#### 2.3.4 MLKL High‐Order Polymerization

Before Meng et al. utilized negative staining electron microscopy to reveal that MLKL forms a tetramer,^[^ ^27^ ^]^ other researchers had also reported that human MLKL forms a tetramer upon activation by native MS analysis on the wild‐type human MLKL oligomer SEC fraction^[^ ^34^ ^]^ and nonreducing gel in HT29 cells,^[^ ^79^ ^]^ respectively. Additionally, some researchers proposed that the formation of the tetramer is not the final stage. They suggested that human MLKL C86 covalent inhibitor NSA could block the polymerization and necroptosis, but could not block the formation of the tetramer.^[^ ^79^ ^]^ Consequently, they argue that the formation of high‐order polymers, rather than merely stopping at the tetramer stage, is necessary for cell necroptosis to occur.^[^ ^61^, ^79^, ^80^ ^]^

Johnston et al. proposed a hypothesis regarding the MLKL high‐order polymers formation. They proved that there is a potential interaction between MLKL and HSP70 and implied that HSP70 played a crucial role in stabilizing MLKL and facilitating its polymerization^[^ ^61^ ^]^ (Figure 4C). This assumption holds that HSP70 can interact with the exposed short hydrophobic peptides in disulfide bond‐dependent tetramers and utilize C574 and C603 of SBD to safeguard and potentially catalyze other cysteines within the tetramer. Subsequently, the HSP70‐bound MLKL tetramer is delivered to the developing MLKL polymer, facilitating the formation of proper disulfide bonds within the polymer.^[^ ^61^ ^]^ The association of HSP70 with the tetramer could also shield it from the reducing activity of Trx1.^[^ ^64^ ^]^ To summarize, in the context of necroptosis, HSP70 functions as a molecular chaperone with a dual role: stabilizing the MLKL protein under normal conditions and facilitating MLKL polymerization by its SBD during necroptosis.

#### 2.3.5 MLKL Membrane Translocation

To explore the regulatory pathways governing the transport of MLKL to the PM, Samson et al. developed an immunofluorescence‐based method to monitor and quantify the activation and movement of endogenous human MLKL at the single‐cell level during necroptosis.^[^ ^35^ ^]^ They pinpointed a Golgi‐microtubule‐actin‐dependent pathway as a critical regulatory mechanism in the execution phase of necroptotic signaling, orchestrating the translocation of MLKL to the PM in epithelial and hematopoietic cell lines. Their research also revealed that MLKL adheres to the identical transportation pathway as Zonula Occludens‐1 (ZO‐1), a protein associated with tight junctions, contributing to the intracellular transport and secretion processes within the cells through interactions with various cytoskeletal components.^[^ ^81^ ^]^ Consequently, during epithelial cell necroptosis, MLKL and tight junction proteins coalesce into distinct clusters at the PM.^[^ ^35^ ^]^

#### 2.3.6 MLKL Endosomal Lysosomal Degradation and “Budding”

Multiple research projects provide evidence for the involvement of MLKL and phosphorylated MLKL in endocytosis, cellular transport, and exocytosis processes. These mechanisms play a role in not only maintaining intracellular MLKL homeostasis but also serving to limit the extent of necroptosis.^[^ ^42^ ^]^ For example, Fan group discovered the co‐localization of MLKL with lipid raft‐associated proteins flotillin‐1 and flotillin‐2 within membrane‐bound MLKL immunoprecipitants. Phosphorylated MLKL can be released through flotillin‐mediated endocytosis, leading to subsequent lysosomal degradation.^[^ ^75^ ^]^ Another research has demonstrated that following activation and oligomerization, MLKL undergoes multi‐mono‐ubiquitination on a minimum of four distinct lysine sites, making it prone to degradation in the proteasome.^[^ ^82^ ^]^ Recent studies by Pradhan et al. have indicated that during necroptosis, C184, C269, and C286 of MLKL undergo palmitoylation, a process that contributes to the binding of MLKL to PM for necroptosis execution, The authors also indicated that inhibition of the acylation of pMLKL leads to enhanced proteasomal degradation.^[^ ^83^ ^]^ All the above processes help control the basal levels of activated MLKL to prevent inadvertent cell death.

MLKL can also translocate to the lysosomal membrane. The aggregation of MLKL on the lysosomal membrane or the induction of aggregation by the NED of MLKL can lead to lysosomal clustering and fusion, ultimately resulting in lysosomal membrane permeabilization (LMP).^[^ ^84^ ^]^ This LMP leads to the rapid release of lysosomal contents into the cytoplasm, causing a significant increase in cathepsin levels, with Cathepsin B (CTSB) being a key factor in subsequent cell death.^[^ ^85^, ^86^ ^]^ Inhibition of MLKL aggregation with NSA can prevent its co‐localization with lysosomes.^[^ ^79^ ^]^ These findings clearly establish the crucial role of MLKL polymerization‐induced lysosomal membrane permeabilization (MPI‐LMP) in the process of necroptosis.^[^ ^84^ ^]^

Furthermore, studies have shown that during necroptosis, ESCRT III components, charged multivesicular body protein 2A (CHMP2A) and CHMP4B, relocate from the cytoplasm to pMLKL near the PM, promoting the release of broken “bubbles” from intact cells to maintain PM integrity.^[^ ^42^, ^87^, ^88^ ^]^ They believed that ESCRT‐III acts to delay the loss of PM integrity induced by active MLKL through the formation of “bubbles”. However, there is controversy over whether bubbles are formed during necroptosis, as “bubbles” may be the result of laser exposure in the microscope. A single‐cell imaging study of necroptotic cells conducted by Samson et al. showed that no “ bubbles ” were observed at the sites where MLKL accumulated.^[^ ^35^ ^]^

#### 2.3.7 MLKL Hotspot Formation

PM binding of MLKL was initially reported driven by electrostatic interactions of positively charged residues within the MLKL's 4HB domain, ultimately triggering the necroptotic process.^[^ ^26^, ^73^, ^89^ ^]^ Hildebrand et al. identified two clusters of residues necessary for the 4HB domain to kill cells using alanine‐ scanning mutagenesis. However, the disruption of membrane localization function by substituting negatively charged residues on α4 helix (E102/K103, R105/D106, E109/E110, and L112–L115) in the first cluster with alanine suggests that membrane binding cannot solely be attributed to the interaction of the poorly conserved basic residues in the MLKL 4HB domain. The second cluster, although, mutations in C‐terminal of α1 helix, N‐terminal of the α2 helix, α1–α2 loop (Y15/E16, K22/R30, and C18/C24/C28), and α3–α4 loop of MLKL do not impede membrane translocation, these mutations significantly attenuate its lethality, implying that beyond membrane translocation, the 4HB domain requires additional functionalities to induce cell death.^[^ ^51^ ^]^ Therefore, emphasizing the positive charge interaction alone is not sufficient to explain the membrane binding and lethality of MLKL. In mutant cells with IPMK and ITPK1 encoding IP kinase, MLKL failed to oligomerize and localize to the membrane despite appropriate RIPK3‐dependent phosphorylation.^[^ ^56^ ^]^ This suggests that necroptosis requires direct binding of highly phosphorylated IP products to positive residues in the NED of MLKL to strongly replace the MLKL autoinhibitory brace helix.^[^ ^56^, ^90^ ^]^ Quarato et al. also proposed a two‐step transition mechanism from low affinity to high affinity for the binding of MLKL to the phosphorylated inositol polar head groups of phosphatidylinositol phosphates (PIPs).^[^ ^78^ ^]^ Moreover, MD simulations conducted by Yang et al. indicated that a cluster of positively charged arginine residues on the C‐terminus of the MLKL autoinhibitory region (R145, R146, R152, and R153) promoted the release of the brace and linker during activation, further suggesting that their interaction with the negative electricity on the membrane may provide additional tension.^[^ ^58^ ^]^

There are various viewpoints on how MLKL destroy the PM. Some believe that it penetrates the PM to form ion channels, leading to leakage of cellular contents and ultimately cell death.^[^ ^32^ ^]^ Some argue that the 4HB may form a cation channel or a pore that allows the flow of cellular contents.^[^ ^91^, ^92^, ^93^ ^]^ A more general hypothesis posits that MLKL directly facilitates the permeabilization of the PM through its partial integration into the lipid bilayer.^[^ ^77^ ^]^ For example, Wang et al. proposed that the oligomeric form of MLKL can bind to negatively charged lipids, including PIPs and cardiolipin, thereby promoting its disruption of membrane integrity.^[^ ^74^ ^]^ Pradhan et al. recently reported that the lipid‐like 24‐carbon acyl tails of MLKL C184, C269, and C286 directly affect the lateral distribution of neighboring lipids in PM, thereby exacerbating membrane permeability during necroptosis.^[^ ^83^, ^94^ ^]^ Ros et al. reported membrane nanopores as the central mechanism of necroptosis.^[^ ^95^ ^]^ Czabotar et al. believed that the mechanism of MLKL pore formation works similarly to the BH3‐in‐groove Bcl‐2 antagonist/killer and BAX homo‐dimers, where the activation leads to the exposure of a hydrophobic surface initiating high molecular weight (HMW) pore‐forming structures.^[^ ^96^ ^]^ Moreover, two alternative models, the carpet model and the toroidal pore model, also suggest possible mechanisms for membrane permeabilization.^[^ ^97^, ^98^, ^99^ ^]^

**Figure 5:**
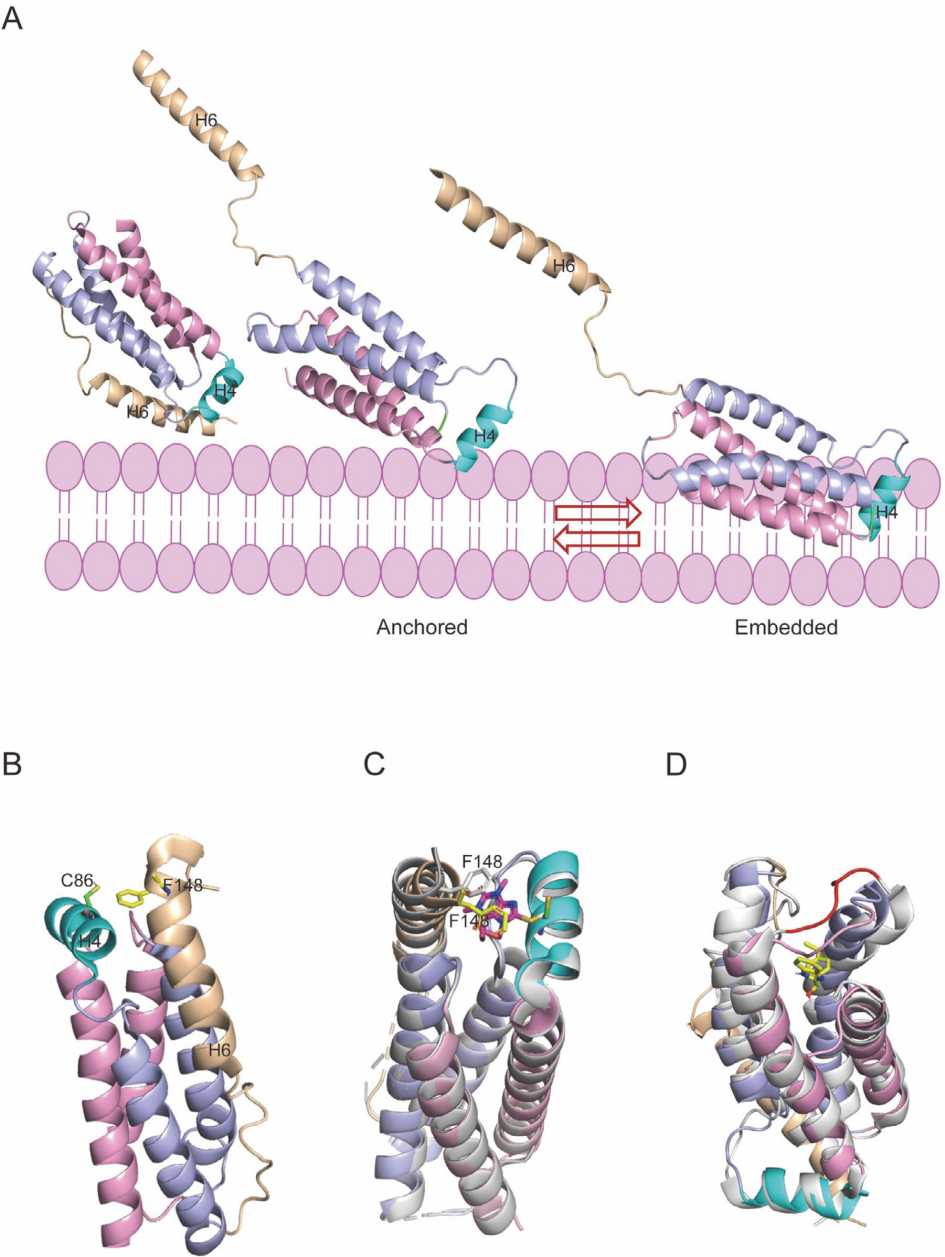
MLKL is directly inserted into the plasma membrane to destroy the permeability of the plasma membrane. Inhibitors acting on the executive domain of MLKL can inhibit the lethality of MLKL. A) Cartoon diagram of possible anchoring and embedding states of MLKL.^[^ ^96^ ^]^ Cyan, helix H4; wheat, brace helix H6. B) The interaction between helix H6 and helix H4 at the apex of the structural domain. The key residues F148 (H6) and C86 (H4) are represented as sticks. C) Alignment of the X‐ray structures of the unliganded MLKL executioner domain (PDB ID: 6ZVO) and MLKL covalently with BI‐8925 (gray; PDB ID: 6ZZ1). The compound is depicted as magenta sticks, with important interacting residues also represented in stick form. D) The NMR structure of the unbound MLKL executioner domain (PDB ID: 6ZLE) and MLKL complexed with Cpd 14 (lightblue, PDB ID: 7NM4) is shown, with Cpd 14 illustrated as yellow sticks.

Yang et al. have recently used a surface‐induced fluorescence attenuation (SIFA) technique to monitor both the axial and lateral movements of a singly labeled MLKL in supported lipid bilayers. Their findings indicated that the NED of MLKL existed in two predominant membrane‐associated states, with the membrane‐insertion region being spatiotemporally distinct from its membrane‐anchoring region^[^ ^100^ ^]^ (**Figure** **5**A). In both the anchored and inserted states, the H4 helix region serves as the anchoring region. Besides, when the NED transitions to the embedded state, H6 detaches from the 4HB. The observations suggest a strong interaction between the membrane and the H4 helical cap region exposed after the separation of H6 from the 4HB, which is essential for the activation of MLKL.^[^ ^25^ ^]^ Yang et al. proposed that the helices H1, H2, H3, and H5 exhibit strong amphipathic properties, featuring hydrophobic surfaces buried inside the domain and highly charged surfaces exposed to the solvent, thereby rendering them unsuitable for insertion into the hydrophobic core of the phospholipid bilayer.^[^ ^77^ ^]^ Only the separation of H6, along with the poorly packed interface between H2 and H5, creates a natural “weak” point within the structure, allowing it to unfold and expose the hydrophobic faces of the helices for insertion into the bilayer. Furthermore, the interaction between H6 and H4 at the apex of the structural domain also contributes to the stability of MLKL, involving the packing of F148 against C86 and other side chains of H4 (Figure 5B). Thus, H6 likely acts as a plug that hinders the membrane permeabilization activity not only by binding to the H2‐ H5 interface but also through its interactions with H4. After MLKL was inserted into PM, Samson et al. employed single‐cell imaging approaches to observe that phosphorylated MLKL steadily co‐accumulates at the PM as heterogeneous micron‐sized hotspots.^[^ ^35^ ^]^

### 2.4 Targeting MLKL Inhibits Necroptosis

Given the significant role of MLKL in necroptosis and inflammation, targeting and inhibiting the checkpoints involved in the MLKL activation process would be beneficial for the prevention and treatment of related diseases. Here, we have compiled a summary of the inhibitors in **Table** **2**.

**Table 2:** Structures of MLKL inhibitors and degraders.

	Compounds structures
ATP‐competitive pseudokinase domain binders	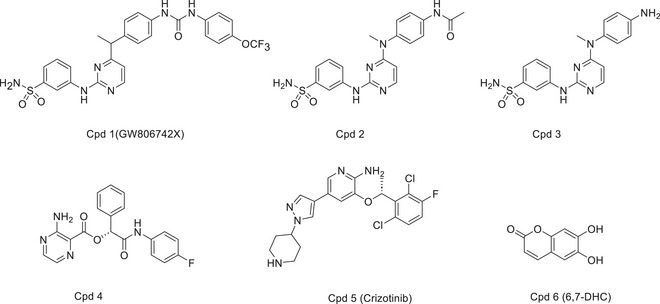
MLKL polymerization and membrane insertion inhibitors	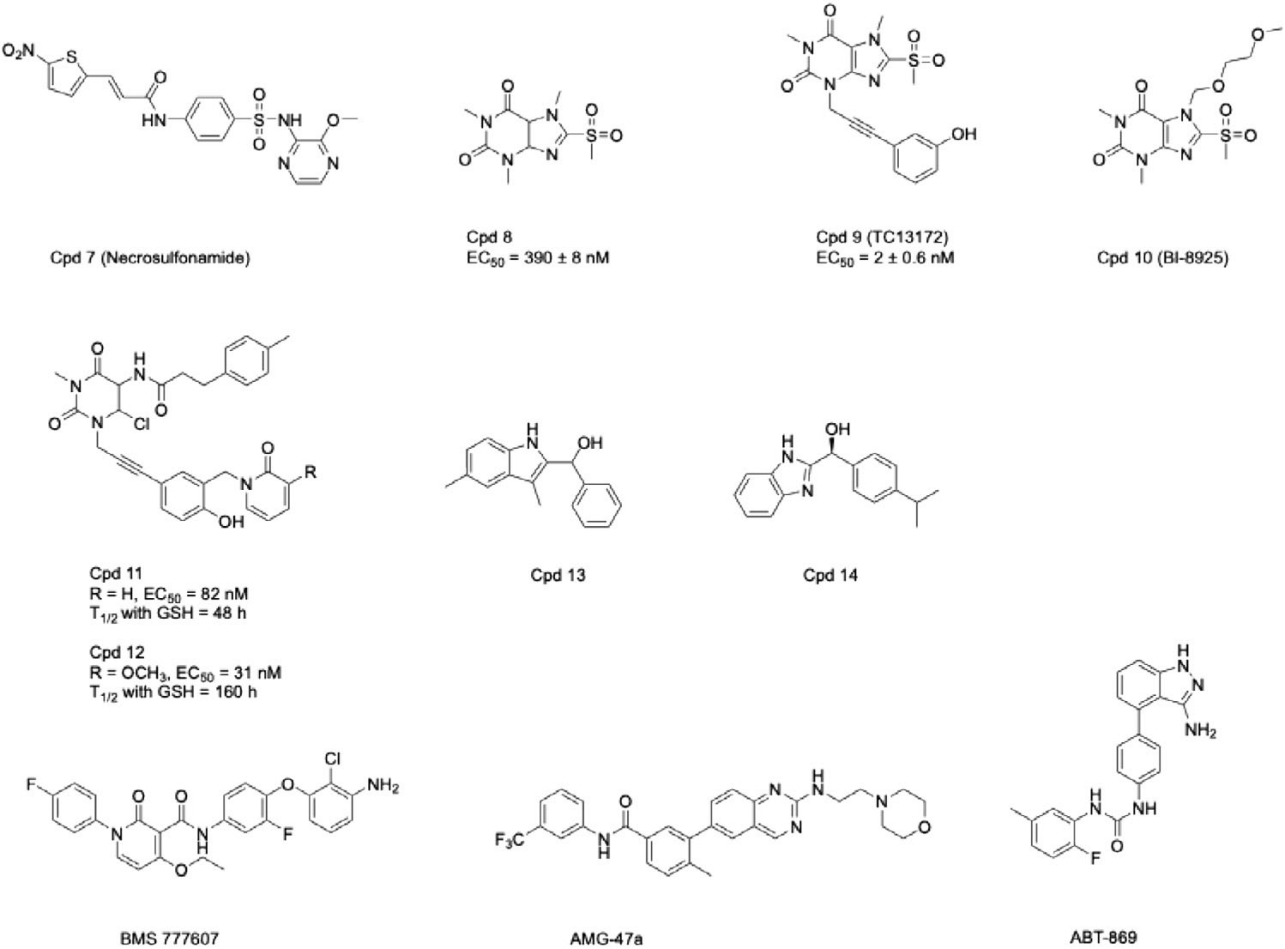
MLKL degraders	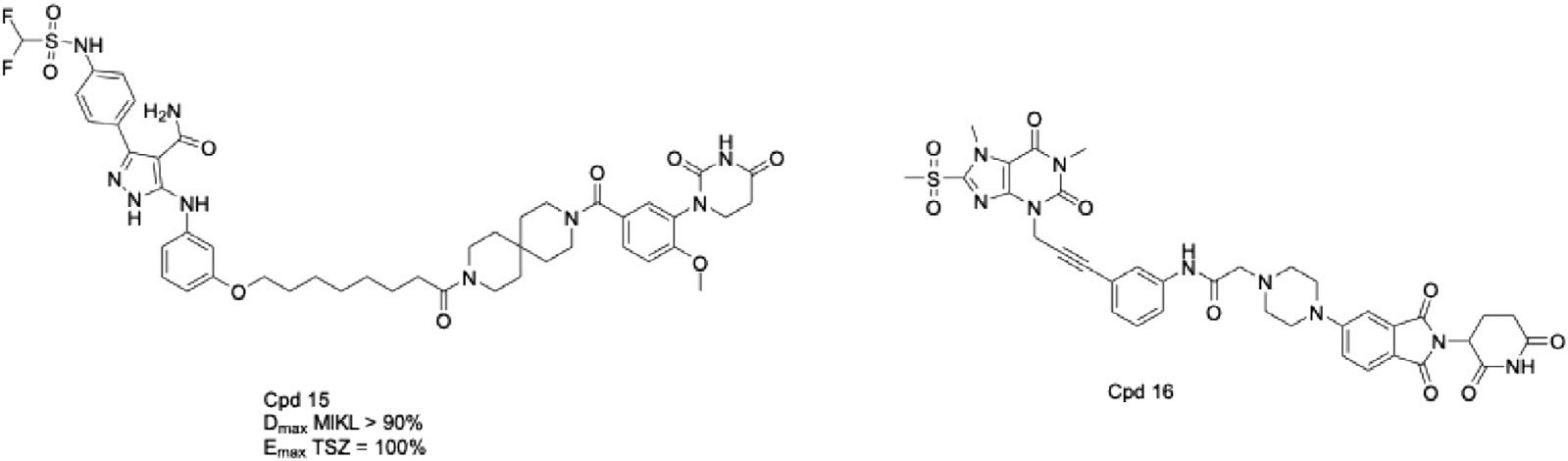

All the inhibitors are currently in the preclinical research stage, except for ABT‐869 (linifanib). As a VEGFR/PDGFR tyrosine kinase inhibitor, ABT‐869 advanced to Phase III trials for hepatocellular carcinoma but was discontinued after failing to demonstrate superior efficacy over sorafenib. It is no longer in active clinical development.

#### 2.4.1 MLKL Pseudokinase Domain Binders

Although MLKL does not possess kinase activity, the pseudokinase domain of MLKL can bind to ATP in a non‐catalytic manner^[^ ^22^, ^39^, ^101^ ^]^ and can be activated by RIPK3. To explore the role of nucleotide binding in MLKL function, Hildebrand et al. screened a library of 367 small molecules using a thermal shift assay against the recombinant mouse MLKL pseudokinase domain, identifying **Cpd 1** (an ATP analogue, also known as **GW806742X**) as an MLKL‐interacting compound.^[^ ^51^ ^]^ The binding of **GW806742X** to the MLKLpseudokinase domain (K_d_ = 9.3 µM) was further verified by surface plasmonic resonance (SPR) analysis.^[^ ^51^ ^]^ However, **GW806742X** did not prevent RIPK3‐mediated phosphorylation, protecting MDFs from TSQ (TNF/Smac/Q‐VD‐OPh) induced necroptosis only at EC_50_ of 1.85 µm, but not at concentrations of > ≈2 µm.^[^ ^69^ ^]^ In addition, GW806742X was associated with side effects such as hepatotoxicity and gastrointestinal reactions. Subsequent studies indicated that the anti‐necroptosis activity of **GW806742X** may be related to its nonspecific binding to RIPK1.^[^ ^69^ ^]^ In brief, without confirmation of MLKL‐mediated specific effects, the anti‐necroptotic effect of these nucleotide analogues cannot be determined. Therefore, they described an MLKL‐selective analogue, **Cpd 4**, and two truncated variants **Cpd 2** and **3** that exhibited improved selectivity against RIPK1. However, neither the **Cpd 4** nor the **Cpds 2** and **3** rescued cells from necroptosis. Similarly, the MLKL‐binding compound **Cpd 5 (Crizotinib)** also failed to protect cells from necroptosis.^[^ ^69^ ^]^ In 2021, Prajapati et al. identified **Cpd 6 (**6, 7‐Dihydroxycoumarin (**6**, **7‐DHC**)) exhibited both preventive and therapeutic effects against oxalic acid‐induced chronic kidney disease in murine models. Through an unbiased high‐content in vitro screening approach, the researchers demonstrated that **6**,**7‐DHC** directly interacts with MLKL and inhibits its phosphorylation. However, the compound was also found to suppress phosphorylation of RIPK1 and RIPK3, suggesting a broader mechanism of action beyond MLKL‐specific inhibition.^[^ ^102^ ^]^ So far, studies of MLKL pseudokinase domain binders appear to have no effect on the necroptotic pathway. However, it cannot be ruled out easily that appropriate ATP analogues of MLKL pseudokinase domain may block the switching mechanism,^[^ ^51^ ^]^ and suitable compounds might induce conformational changes in MLKL, thereby reducing the accessibility of the activation loop and preventing MLKL phosphorylation.^[^ ^69^ ^]^

#### 2.4.2 Inhibition of MLKL Oligomerization and Membrane Insertion

The formation of MLKL oligomers primarily depends on interactions of NED and the brace region. The first identified MLKL inhibitor **Cpd 7 (NSA)** was a compound that covalently bound to the NED H4 at position C86 through a Michael receptor.^[^ ^1^ ^]^ Subsequent studies have shown that **NSA** blocked the oligomerization of MLKL tetramers and subsequent cell death (IC_50_ < 1 µm).^[^ ^79^ ^]^ Nevertheless, the C86S mutant and mouse MLKL lacking the corresponding cysteine still formed tetramers and polymers, suggesting that C86 itself was not essential for polymer formation. A plausible explanation is that conjugated **NSA** occupied the space between tetramers, preventing them from coming close enough to polymer. Moreover, **NSA** does not completely block tetramer formation.^[^ ^79^ ^]^ **NSA** has also been reported to cross‐link the C86 of human MLKL with C32 of the Trx1, inhibiting the disulfide bonds formation and polymerization of in MLKL in vitro.^[^ ^64^ ^]^ In addition to inhibiting necroptosis, **NSA** can also covalently bind to the key cysteine residues C191/C192 of GSDMD, thereby inhibiting the activity of GSDMD and the pyroptosis.^[^ ^103^ ^]^

Following the discovery of **NSA**, Wang et al. identified **Cpd 8**, a xanthine covalent inhibitor of C86 (EC_50_ = 390 ± 8 nm) through a high‐throughput screening.^[^ ^104^ ^]^ After optimization, **Cpd 9 (TC13172)** was obtained with an EC_50_ value of 2 nm.^[^ ^104^ ^]^ The xanthine classical inhibitor **Cpd 10 (BI‐8925)** also inhibited Jurkat and U937 cell necroptosis with IC_50_ values of 541 and 271 nm, respectively.^[^ ^105^ ^]^ Rübbelke's X‐ray (PDB ID: 6ZZ1) and NMR co‐structures revealed that **BI‐8925** stabilized the interaction between the auto‐inhibitory H6 and the 4HB by stacking to Phe148 (Figure 5C), and the importance of this interaction was further validated by the F148A mutant.^[^ ^106^ ^]^ Cui et al. created highly potent compounds **11** and **12** containing an uracil core by optimizing **TC13172**.^[^ ^107^ ^]^ These compounds partially impeded MLKL oligomerization but markedly hindered MLKL membrane translocation, with less potential for off‐target effects and cytotoxicity.^[^ ^107^ ^]^ In summary, these C86 covalent inhibitors inhibit MLKL oligomerization, membrane binding, and insertion state. Together, these results seem to confirm the conclusion that C86 itself does not affect necroptosis, but that the C86 covalent inhibitor on H4 appears to play the same role as the phosphorylation of the neighboring residue S83.^[^ ^67^ ^]^ In addition, the lack of target cysteine in rodents homologous MLKL makes such inhibitors unsuitable for rodent disease models. Therefore, the covalent mechanism limits their applicability as therapeutic agents.

Rübbelke et al. identified a non‐covalent MLKL NED inhibitor **Cpd 13** using protein detection NMR.^[^ ^108^ ^]^ Affinity‐enhanced **Cpd 14**, obtained through scaffold hopping, locating its iso‐propyl‐phenyl group toward the hydrophobic core adjacent to H2 and H5 (from NMR structure, PDB ID: 7NM4) (Figure 5D). The NMR relaxation experiments and X‐ray crystal structures indicated that Cpd14 fully occupied flexible structure and a partially open conformation formed by H2‐ H3 loop (PDB ID: 6ZVO)^[^ ^106^ ^]^ (Figure 5D). However, despite their ability to hinder the binding of monomeric detergent molecules, these compounds seem insufficient in stabilizing the 4HB or the auto‐inhibitory attachment of H6 to prevent oligomerization.^[^ ^108^ ^]^

In addition to the inhibitors that directly act on MLKL, TAM kinase inhibitor **BMS‐777607** also controls MLKL oligomerization but does not control phosphorylation or membranal translocation.^[^ ^62^ ^]^ The HSP70 inhibitor NBC1 inhibits the interaction of SBD and MLKL NED to promote MLKL polymerization and stabilization but does not inhibit tetramerization.^[^ ^61^, ^109^ ^]^ The lymphocyte‐specific protein tyrosine kinase (Lck) inhibitor **AMG‐47a**, which interacts with both RIPK1 and RIPK3 and targets a mechanism downstream of MLKL dimerization, perhaps by inhibiting MLKL oligomerization or by inhibiting MLKL translocation to membranes.^[^ ^51^, ^53^ ^]^ Pierotti et al. screened identified the vascular endothelial growth factor receptor (VEGFR) and platelet‐derived growth factor receptor (PDGFR) tyrosine kinase inhibitor, **ABT‐869** (Linifanib), which also targets RIPK1 to inhibit the cell death of self‐activated MLKL mutant cells.^[^ ^110^ ^]^

#### 2.4.3 Inhibition of MLKL Membrane Transport and Membrane Binding

In the necroptosis effector stage, MLKL is transported to PM through Golgi‐microtubule‐actin‐dependent mechanisms. The combination of Nocodazole, Cytochalasin B, and Brefeldin A (NCB) provided a highly effective strategy to block PM disruption at any stage of necroptosis. It delays the binding of MLKL to the membrane but does not interfere with the binding of MLKL to the membrane, ultimately reducing the size of the hot spot.^[^ ^35^ ^]^ Petrie et al. previously developed monobodies, including Mb33 (from PDB ID: 6UX8) and Mb37, to investigate the precise checkpoints during necroptosis. These two monobodies bind to the H6 helix and the adjacent loop of human MLKL with K_d_ values of 141 ± 12 nm and 170 ± 21 nm,.^[^ ^25^ ^]^ Therefore, they are unlikely to prevent MLKL tetramerization but could impede the release of the 4HB domain from the brace region and subsequent membrane binding.^[^ ^27^ ^]^ Liu et al. demonstrated that repulsive guidance molecules b (RGMb) inhibited MLKL membrane translocation or binding without affecting phosphorylation and oligomerization, providing protection against acute kidney injury (AKI) by inhibiting MLKL membrane association and necroptosis in proximal tubular cells.^[^ ^111^ ^]^

#### 2.4.4 Inhibition of MLKL Stability

Skp2 (S‐phase kinase‐associated protein 2) is an E3 ligase and an essential element of the SCF (Skp1‐Cullin1‐F‐box) type of E3 ubiquitin‐ligase complexes, Zhou et al. previously discovered to be responsible for ubiquitinating and subsequently degrading MLKL, and they believed that Skp2 partially contributes to the development of cisplatin resistance in non‐small cell lung cancer (NSCLC) cells.^[^ ^112^ ^]^ The authors also reported that the Skp2 inhibitor SZL P1‐41 can disrupt the Skp2‐related binding and degradation of MLKL in A549 cells.^[^ ^112^ ^]^ However, the A549 cell line, as a human NSCLC cell line, is not susceptible to necroptosis stimulation. Therefore, in this case, targeted NSCLC cells may not necessarily have therapeutic value. In addition to Skp2, Rathje et al. achieved the degradation of MLKL by another E3 ubiquitin ligase cereblon (CRBN) through the PROteolysis TArgeting Chimera (PROTAC) technique. These PROTAC molecules used the high‐affinity pyrazole carboxyamide as an MLKL ligand and lenalidomide as a CRBN ligand. Among them, **Cpd 15** was the most effective MLKL degrader, achieving almost complete (89%) degradation of MLKL in a dose‐dependent manner at 10 µm and 60% at 1 µm. However, the author did not evaluate the MLKL degradation activity of **Cpd 15** in animals.^[^ ^113^ ^]^ Li et al. also developed a series of covalent PROTACs by linking and optimizing a theophylline derivative, ultimately identifying **Cpd 16** (**MP‐11**) as a potent MLKL degrader. Since **Cpd 16** covalently binds to the C86 of human MLKL, which is not conserved in mouse MLKL, the authors believed that the ability of **Cpd 16** to effectively degrade MLKL in the mouse xenograft model does not necessarily indicate that the compound has in vivo degradative activity. Consequently, this covalent characteristic limits the broader applicability of this compound in research.^[^ ^114^ ^]^

#### 2.4.5 Other Aspects

In 2018, a cell‐based screening revealed that BET inhibitors protected cells from necroptosis in a TSZ‐induced cell death model. Mechanistic studies revealed that BRD4, IRF1, p‐TEFb, and RNA polymerase II formed a transcription complex to regulate the expression of MLKL, while BET inhibitors interfere with the formation of the transcriptional complex and downregulate MLKL expression. Therefore, BET inhibitors, such as JQ‐1, have shown promising therapeutic effects in models of necroptosis related diseases.^[^ ^115^ ^]^

## 3 Discussion and Outlook

We have comprehensively summarized the activation checkpoints of necroptosis at the cellular and protein levels. However, regarding the occurrence and location of necroptosis in vivo, some research progress has also been made. For instance, Chiou/Samson provides automated immunohistochemistry protocols to detect core necroptosis regulators – Caspase‐8, RIPK1, RIPK3, and MLKL – in formalin‐fixed mouse and human tissues.^[^ ^116^ ^]^ Tanzer et al. combined phosphoproteomic time course experiments of TNF‐induced cell death with subcellular localization analysis through mass spectrometry, providing detailed insights into phosphorylation events.^[^ ^117^ ^]^ An increasing number of research suggests that RCD may occur simultaneously in certain circumstances. It is challenging to clearly define which type of cell death predominates in these cases. However, the expression of MLKL and subsequent cell lysis are critical determinants for the concurrent occurrence of RCD. Therefore, gaining a better understanding of all functions of MLKL could aid in devising control strategies in many other physiological and pathological processes. We have compiled literature on the MLKL structures and the inhibitors from 2012 to the present. While various aspects of MLKL activation have been reported, none have been determined based on full‐length human MLKL. The conclusions drawn from these studies still contain many unknown aspects. For example, Does MLKL form distinct oligomeric states in response to varying stimuli? How does the MLKL oligomer localize the 4HB to interact with the PM? How does the membrane itself promote higher‐order oligomerization of MLKL? How do lipid interactions at the PM influence its pore‐forming activity? Resolving these questions will require integrative approaches, combining structural biology, super‐resolution microscopy, and computational modeling. Additionally, the potential crosstalk between MLKL and other cell death executors (e.g., gasdermins in pyroptosis) remains underexplored. Could simultaneous inhibition of multiple pore‐forming proteins yield better therapeutic outcomes? Finally, the development of biomarkers to stratify patients who would benefit from MLKL‐targeted therapies is crucial for clinical translation.

Although the research results of **GW806742X** and other MLKL pseudokinase domain binders have been unsatisfactory, the successful approval of other pseudokinase domain inhibitors^[^ ^118^ ^]^ has brought hope for the feasible prospect of targeting the conformational changes and interactions of MLKL pseudokinase as a drug discovery strategy. In addition, the development of MLKL pseudokinase domain inhibitors can also draw inspiration from the research conducted by Ros et al. regarding human MLKL isoforms (hMLKL1, UniProt: Q8NB16‐1, hMLKL2, UniProt: Q8NB16‐2, and hMLKL0: the potential existence of an alternative splicing variant containing five additional amino acids in the Hc (462‐471) of hMLKL1).^[^ ^119^ ^]^ Their authors found that the interaction between Hc and a previously unrecognized hydrophobic groove (residues from the 4HB, brace, and pseudokinase domains) is essential for necroptosis. hMLKL0 can inhibit the Hc/groove interaction and is an isoform that inhibits necroptosis mediated by hMLKL1.^[^ ^119^ ^]^ Therefore, research into potential inhibitors targeting the Hc/groove of human MLKL may guide the development of allosteric MLKL inhibitors for the treatment of human diseases. However, it is undeniable that the notable absence of MLKL‐targeting drugs in clinical development reflects complex biological and pharmacological challenges. For examples, MLKL's dual physiological roles in antibacterial defense and intestinal barrier maintenance, coupled with the vascular and immunological defects observed in MLKL‐deficient mice^[^ ^40^, ^120^, ^121^ ^]^ raise significant safety concerns regarding systemic inhibition. Molecular design is further complicated by structural divergence in the 4HB domain between species^[^ ^36^ ^]^ and potential compensatory activation of apoptosis/pyroptosis upon MLKL inhibition.^[^ ^122^ ^]^ These translational hurdles highlight the need for innovative approaches such as tissue‐specific delivery systems or combination therapies targeting parallel cell death pathways.

Further research is needed to elucidate the structural and functional nuances of MLKL across different cellular contexts. Cryo‐EM and X‐ray crystallography studies have provided insights into MLKL's autoinhibited and activated states, but how post‐translational modifications (e.g., ubiquitination, phosphorylation at alternative sites) or interacting proteins modulate its function remains unclear. For example, MLKL's role in non‐necroptotic processes, suggests pleiotropic functions that could influence therapeutic outcomes. Moreover, cell‐type‐specific differences in MLKL regulation warrant investigation. For instance, why do some cancer cells exhibit MLKL‐dependent necroptosis while others evade it? Does MLKL's subcellular localization (e.g., nuclear vs cytoplasm) affect its pro‐death or pro‐survival functions? Addressing these questions will require advanced models, such as organoids or patient‐derived xenografts, to capture the heterogeneity of MLKL behavior in vivo. To fully unravel these processes, a collaborative and interdisciplinary approach, involving the concerted efforts of structural biologists alongside researchers from diverse yet complementary disciplines, is paramount. This underscores the indispensable nature of interdisciplinary research in advancing our knowledge of MLKL and its role in disease.

## Conflict of Interest

The authors declare no conflict of interest.
